# Review the progression of ovarian clear cell carcinoma from the perspective of genomics and epigenomics

**DOI:** 10.3389/fgene.2023.952379

**Published:** 2023-02-16

**Authors:** An Tong, Xiangjie Di, Xia Zhao, Xiao Liang

**Affiliations:** ^1^ Department of Gynecology and Obstetrics, Key Laboratory of Obstetrics and Gynecologic and Pediatric Diseases and Birth Defects of Ministry of Education, Development and Related Diseases of Women and Children Key Laboratory of Sichuan Province, West China Second University Hospital, Sichuan University, Chengdu, Sichuan, China; ^2^ Clinical Trial Center, NMPA Key Laboratory for Clinical Research and Evaluation of Innovative Drugs, West China Hospital of Sichuan University, Chengdu, Sichuan, China

**Keywords:** ovarian clear cell carcinoma, genomics, epigenomics, gynecological oncology, review

## Abstract

Ovarian clear cell carcinoma (OCCC) is a rare subtype of epithelial ovarian cancer with unique molecular characteristics, specific biological and clinical behavior, poor prognosis and high resistance to chemotherapy. Pushed by the development of genome-wide technologies, our knowledge about the molecular features of OCCC has been considerably advanced. Numerous studies are emerging as groundbreaking, and many of them are promising treatment strategies. In this article, we reviewed studies about the genomics and epigenetics of OCCC, including gene mutation, copy number variations, DNA methylation and histone modifications.

## 1 Introduction

Ovarian cancer is one of the most common malignancies in the female reproductive system, accounting for 2.5% of all malignancies among females (Torre et al., 2018). The prognosis of ovarian cancer is poor, and the 5-year survival rate is only 47.6%, largely driven by late-stage diagnoses (Eisenhauer, 2017). The histological and pathological type of ovarian cancer is an important factor in the prognosis of patients, as well as one of the major pieces of evidence for chemotherapy. Epithelial ovarian cancer (EOC) is the most common subtype of ovarian cancer, accounting for approximately 90% of ovarian cancers. According to the histological type, it is now widely accepted that EOC can be further subdivided into five main histological types (Lheureux et al., 2019). Among them, high-grade serous ovarian cancer (HGSOC) is the predominant subtype, accounting for 70%–80% of EOCs (Barnes et al., 2021). Other rarer subtypes include low-grade serous (<5%), endometrioid (10%), clear cell (6%), mucinous (6%) and other rare or undefined cancers (Lu and Chen, 2014).

**FIGURE 1:**
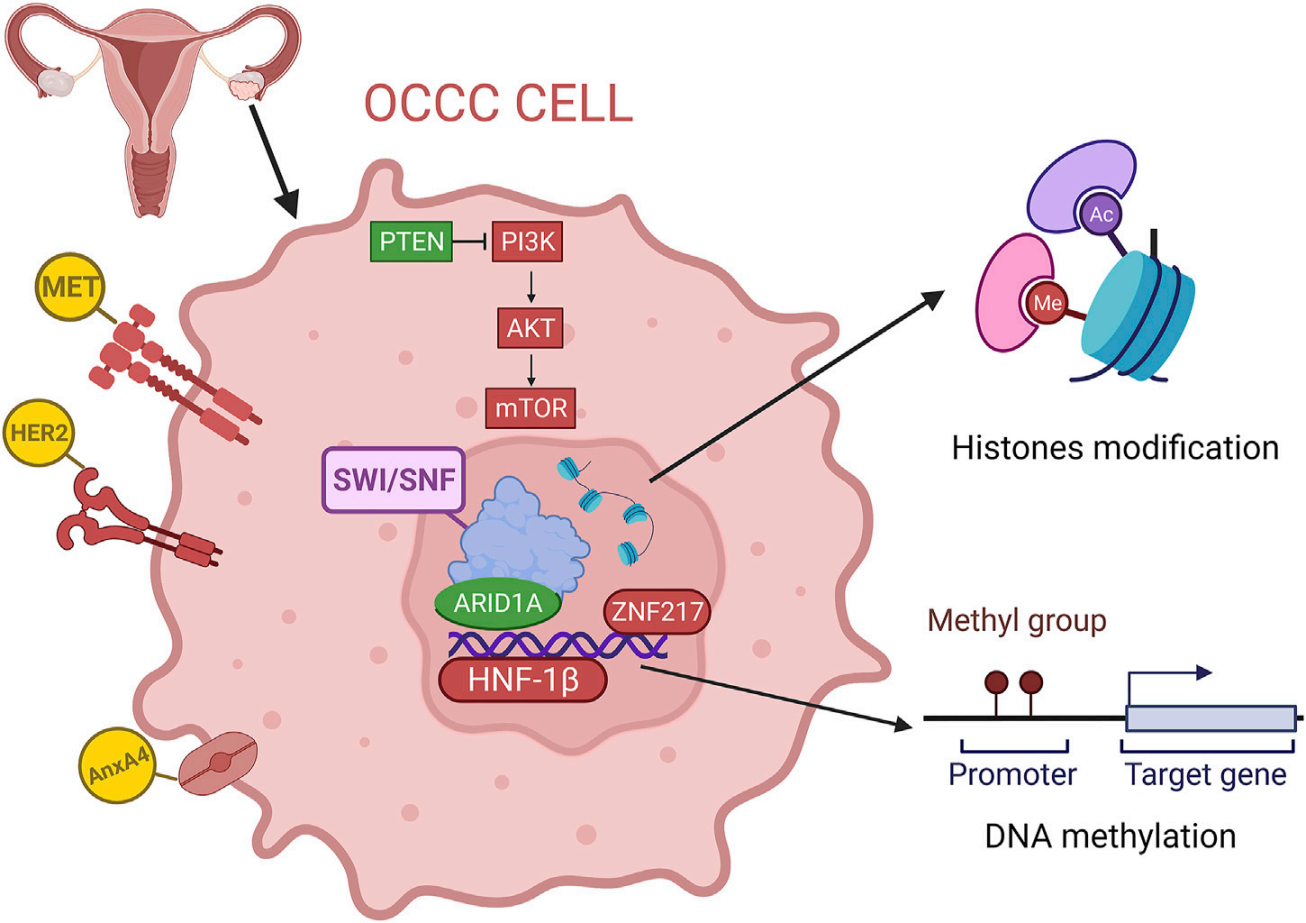
Overview of the genomics and epigenetics of OCCC, including gene mutation, copy number variations, DNA methylation and histone modification. (red for overexpression or amplification, green for deletion).

Ovarian clear cell carcinoma (OCCC) was first named mesonephroma by Schiller in 1939 because it was thought to originate from the mesonephric gland, similar to kidney cancer (Saavedra and Sandow, 1968). Then, it was recognized as a distinct histologic subtype of ovarian cancer and termed ovarian clear cell carcinoma by the World Health Organization in 1973 (Korenaga et al., 2020). As the rare subtype of EOC, OCCC has unique histopathological characteristics and specific biological and clinical behaviors compared with other types of ovarian cancers (Kishikawa et al., 1995). OCCC is most frequently discovered in perimenopausal women and comprises approximately 5%–10% of ovarian cancers in North America and Europe (Torre et al., 2018). In many Asian countries, especially in Japan, the proportion is significantly higher at 15%–25%, probably due to genetic and environmental differences (Sung et al., 2014; Schnack et al., 2016). Most OCCC patients present with a unilateral large ovarian mass, which is more likely to be diagnosed early in the disease progression (57%–81% in stage I/II) and has a higher overall survival rate than contemporary HGSOCs (Anglesio et al., 2011). In contrast, OCCC is considered to have a drug-resistant phenotype, as the response rate to traditional paclitaxel plus carboplatin chemotherapy was lower than that of the other histologic types, ranging from 22% to 56% (Ho et al., 2004; Jin et al., 2014). Therefore, OCCC has a higher risk of relapse, and advanced OCCC (stage III/IV) has a poorer prognosis than contemporary HGSOC, with a 5-year survival rate of approximately 20% (Winter et al., 2007; del Carmen et al., 2012). Numerous studies have found that OCCC is closely associated with endometriosis and is thought to be a malignant transformation of benign ectopic endometrium on the ovary (Pearce et al., 2012; Gadducci et al., 2014; Suda et al., 2020; Shin et al., 2021). Patients with endometriosis have a ninefold increased risk of ovarian cancer (Kobayashi et al., 2007). In addition, studies have shown that tubal ligation can reduce the incidence of OCCC, which may result from preventing the formation of endometriosis lesions due to the countercurrent of menstrual blood (Kurian et al., 2005). On the other hand, the expansion of genome-wide technologies has revealed that the molecular characteristics of OCCC are distinctly different from those of other subtypes. As we known, HGSOC is recognized by near-ubiquitous *TP53* (tumor protein 53) mutation and/or *BRCA1/2* (breast cancer susceptibility gene 1/2) mutation, somatic and/or germline mutations in genes involved in genome-wide copy-number variation and homologous recombination repair (Kurman and Shih Ie, 2010; Cancer Genome Atlas Research, 2011; Huang et al., 2018; Barnes et al., 2021). However, existing studies have found that the pathogenesis of OCCC is similar to endometrial cancer, such as inactivation of tumor suppressor genes such as *PTEN* (phosphatase and tensin homologue) and *ARID1A* (AT-rich interactive domain 1A), activation of the PI3K/AKT/mTOR (phosphoinositide-3 kinase/protein kinase-B/mammalian) signal pathway, and high microsatellite instability (MSI) (Ueda et al., 2005; Gounaris et al., 2011; Xiao et al., 2012; Worley et al., 2015; Willis et al., 2017). With the development of next-generation sequencing, our knowledge about tumorigenesis and the mechanisms of tumor cell proliferation, immune avoidance and antitumor therapy has increased (Munoz-Galvan and Carnero, 2021). In this review, we summarized recent advances in the molecular characteristics of OCCC from the perspective of genomics and epigenomics (Figure 1).

## 2 Genomics of ovarian clear cell carcinoma

Whole-genome sequencing (WGS) and whole-exome sequencing (WES) are the two major approaches in genomic studies (Xiao et al., 2021). WGS or WES have been used to explore the genomic alterations involved in tumorigenesis that have been summarized in this review, such as gene mutations, copy number alterations and structural variants (Li et al., 2020; Zelli et al., 2020).

### 2.1 Loss of *ARID1A* and BAF250a expression

AT-rich interactive domain 1A (*ARID1A*), located on human chromosome 1p35.3, is a frequently mutated gene locus in many tumors, including OCCC, endometrial cancer, ovarian endometrioid cancer, gastric cancer, colorectal cancer and pancreatic cancer (Guan et al., 2011a; Wang et al., 2011; McConechy et al., 2012; Kadoch et al., 2013). *ARID1A*, encoding BRG1-associated factor 250a protein (BAF250a), is the most frequently mutated gene in OCCC, seen in 46%–57% of cases (Sato et al., 2018; Yano et al., 2019). It was reported that *ARID1A* mutations and loss of BAF250a expression were often evident in OCCC tumors and contiguous atypical endometriosis instead of distant endometriotic lesions (Wiegand et al., 2010). Furthermore, studies discovered that *ARID1A* mutations and loss of BAF250a protein accumulated in a stepwise manner during the transformation process from benign endometriosis through atypical endometriosis to OCCC, suggesting that *ARID1A* mutation occurred as a very early event in OCCC development (Yamamoto et al., 2012a; Xiao et al., 2012). Choi et al. classified OCCCs according to *ARID1A* expression status and found that *ARID1A*-mutated OCCCs exhibited distinct immunohistochemical features from the others, suggesting a different underlying molecular event during carcinogenesis (Choi et al., 2017). Additionally, *ARID1A*-mutated OCCC has shorter progression-free survival (PFS) than *ARID1A*-unmutated OCCC, but there was no significant difference in overall survival (OS) (Katagiri et al., 2012; Itamochi et al., 2015; Ye et al., 2016). However, Katagiri et al. (2012) revealed that among OCCC patients undergoing platinum-based chemotherapy, patients with *ARID1A* had shorter PFS and OS and were more resistant to chemotherapy. Moreover, Lyu et al. (2016) used siRNA to effectively silence the expression of *ARID1A* in the human OCCC cell line ES2 and found that cell apoptosis was decreased and its sensitivity to cisplatin chemotherapy was reduced. Both studies indicated that *ARID1A* loss in OCCC was a negative prognostic factor for platinum chemotherapy patients.

**FIGURE 2:**
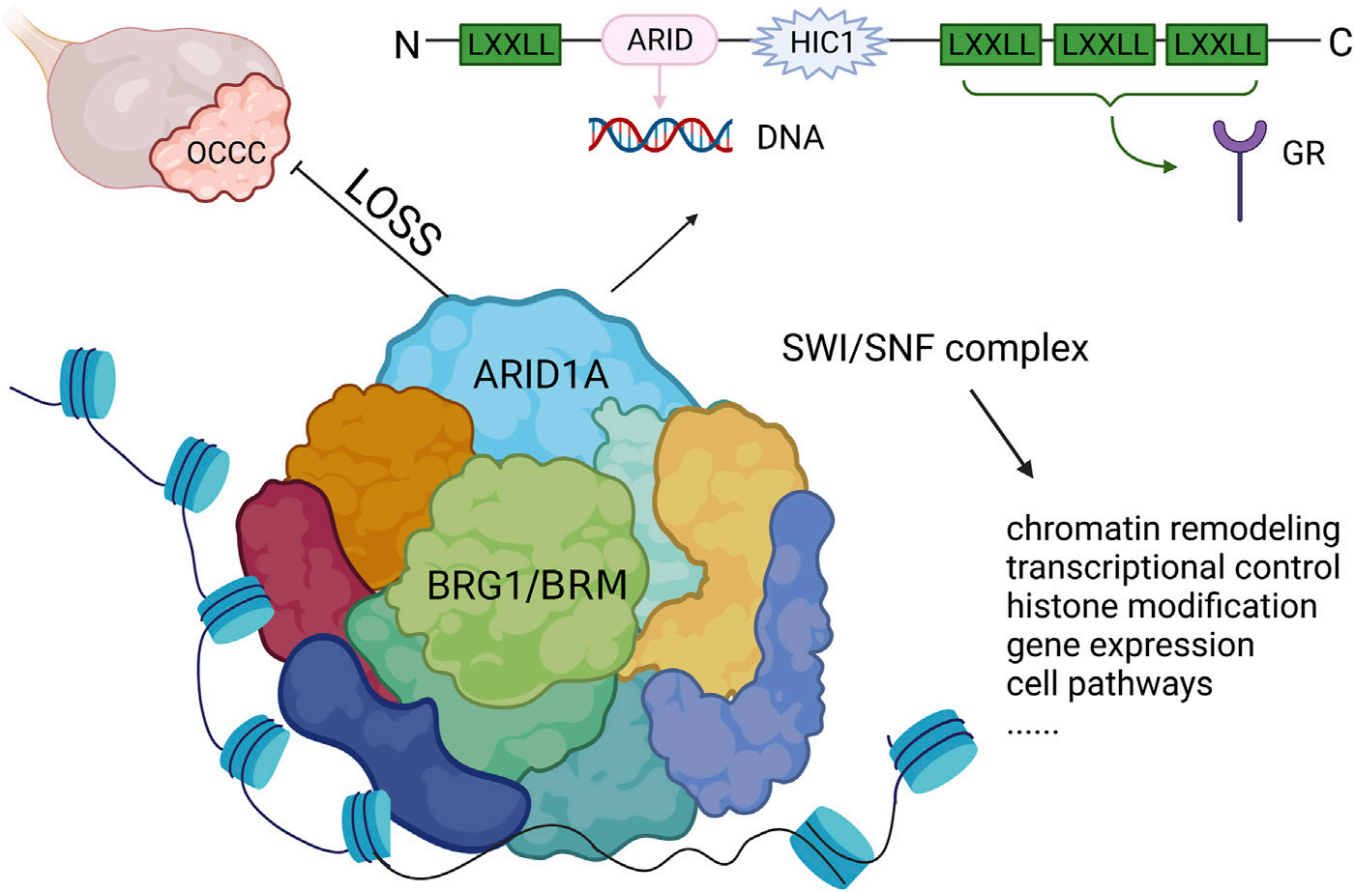
The structure of *ARID1A* and its role in OCCC. *ARID1A* mutation leads to the loss of expression and inhibits the function of SWI/SNF complex, which drives tumor malignancy. LXXLL, Leucine-x-x-leucine-leucine motif; ARID, AT-rich interactive domain; HIC1, hypermethylated in cancer 1 domain; GR, glucocorticoid receptor.

Mutations in *ARID1A* are often heterozygous non-sense or frameshift variants that are not enriched in hotspot sites (Jones et al., 2010; Wiegand et al., 2010). Studies discovered that both ARID1A alleles were affected through two mutations which were presumably biallelic or through either a mutation in one allele and loss of heterozygosity of the other allele. Thus, both homozygous and heterozygous mutations lack *ARID1A* protein expression (Caumanns et al., 2018a). BAF250a, encoded by *ARID1A*, has an N-terminus containing a LXXLL motif and a DNA binding domain consisting of 100 amino acids, which binds DNA sequences in a non-specific manner. Meanwhile, there are also three LXXLL motifs at the C-terminus, which constitute a glucocorticoid receptor binding domain that is involved in regulating the transcription and expression of several genes important in tissue repair, embryo development, cell cycle regulation, cell proliferation, aging, apoptosis and tumorigenesis (Nie et al., 2000) (Figure 2). The *ARID1A* protein has the ability to bind to proteins or DNA and regulate gene transcription and expression and has an important role in tissue repair, embryo development, cell cycle regulation, cell proliferation, aging, apoptosis and tumorigenesis (Nie et al., 2000; Jones et al., 2010; Nakonechny and Gilks, 2016). In addition, *ARID1A* protein is a key component of SWI/SNF (mating-type switching/sucrose non-fermenting), an ATP-dependent chromatin remodelling complex. This complex is necessary for the function of DNA repair proteins, such as *p53, BRCA1, GADD45* (growth arrest and DNA damage gene 45) and Fanconi anemia protein, and therefore play an important role in DNA damage repair (Ribeiro-Silva et al., 2019). A subsequent study showed that the SWI/SNF complex is the most frequently mutated chromatin regulatory complex (CRC) in human cancer with a variety of mutation patterns, similar to *TP53* (Kadoch et al., 2013). The SWI/SNF complex is also increasingly recognized as a tumor-inhibiting complex whose subunit mutations occur in a variety of malignancies, including non-small-cell lung cancer, bladder transitional cell carcinoma, pancreatic cancer and breast cancer (Gui et al., 2011; Zhu et al., 2015; Takao et al., 2017; Naito et al., 2019).

Studies have demonstrated that *ARID1A* mutations often coexist with *PTEN* or *PIK3CA* mutations in tumors, suggesting a synergistic role in tumorigenesis (Yamamoto et al., 2012a). Guan et al. found that loss of *ARID1A* alone is insufficient for tumor initiation, but additional genetic alterations were needed, such as *PTEN* deletion, to drive tumorigenesis (Guan et al., 2014). A recent study involving ten institutions across five countries revealed that *ARID1A* mutations were present almost exclusively in OCCC while *TP53* mutations were in non-OCCC by examining genome-wide tumor methylation and gene expression (Cunningham et al., 2022). Another study also found that mutations in the *ARID1A* and *TP53* genes were irrelevant in OCCC- and endometriosis-associated ovarian cancers. *ARID1A* mutation did not affect the binding rate of p53 protein to cyclin-dependent kinase inhibitor 1A (*CDKN1A*) gene and Sma and Mad-related gene (*SMAD3*) promoters, but both *CDKN1A* and *SMAD3* protein expression were significantly decreased. At the same time, *ARID1A* could not induce the expression of *CDKN1A* and *SMAD3* in cell lines with *TP53* knockout or after RNA interference. Finally, evidence was obtained that the ARID1A/BRG1 complex interacted directly with p53, which synergistically regulated the transcription of *CDKN1A* and *SMAD3* and negatively regulated the cell cycle. These results indicated that both *ARID1A* and *TP53* were necessary to inhibit tumor growth, and mutations in either of them inactivated the complex and resulted in the loss of tumor suppression (Guan et al., 2011b). Zeng et al. discovered that overexpression of *ARID1A* in glioma cells reduced the expression of pAKT and pS6K and arrested the cell cycle in G1/S phase through the PI3K-AKT signaling pathway, leading to inhibition of cell proliferation and induction of apoptosis in glioma cells (Zeng et al., 2016). In addition, *ARID1A* can recruit the protein MSH2 (MutS Homolog 2) into chromatin during DNA replication, which plays an important role in mismatch repair. Loss of *ARID1A* is associated with mismatch repair defects, high mutant load, high PD-L1 expression, high MSI, and an increased number of tumor-infiltrating lymphocytes in many cancers (Caumanns et al., 2018a). Kurota et al. found that *ARID1A* mutation was associated with high PD-L1 in OCCC but not in other histologic subtypes of ovarian cancer (Kuroda et al., 2021). It was also reported that mice with *ARID1A* mutations were more sensitive to PD-L1 immune checkpoint blockade in ovarian and colorectal cancer models than wild-type mice, indicating that *ARID1A* deficiency could be coordinated with immune checkpoint blockade therapy (Shen et al., 2018). However, a small study on OCCC patients found higher expression of PD-L1 and more tumor-infiltrating lymphocytes in tumors with high MSI, but the relationship with ARID1A mutation has not been studied (Howitt et al., 2017). Nevertheless, high MSI is relatively low in OCCC (10%–14%); thus, the proportion of OCCC tumors with both high MSI and *ARID1A* mutations will be lower (Cai et al., 2004; Howitt et al., 2017). Therefore, exploring immunotherapy in *ARID1A*-mutated OCCC may be suitable for only a small number of patients. Interestingly, studies in recent years have found that *ARID1A* deficiency is an important factor for targeted therapies towards synthetic lethality in OCCC (Takahashi et al., 2021). *HDAC2, HDAC6, BRD2* (bromodomain containing protein 2), *CCNE1* (cyclin E1), and *YES1* (v-YES-1 Yamaguchi sarcoma viral oncogene homolog 1) inhibitors were found to specifically cause the synthetic lethality of *ARID1A* mutant OCCC, providing potential options for the targeted therapy of OCCC (Miller et al., 2016; Caumanns et al., 2018a; Kawahara et al., 2021).

All the above results indicate that *ARID1A* is a potential tumor suppressor gene that is closely related to the tumorigenesis and chemotherapy resistance of OCCC. Therefore, new therapeutic strategies, such as *ARID1A* mutations, in OCCC might improve chemotherapy sensitivity, prolong survival, and even prevent malignancy from progressing from endometriosis to OCCC.

### 2.2 Amplifications and/or mutations of the PI3K/AKT/mTOR signaling pathway

The phosphoinositide-3 kinase/protein kinase-B/mammalian target of rapamycin (PI3K/AKT/mTOR) signaling pathway not only plays an important role in cell physiology but is also abnormally activated in a variety of human cancers, leading to cell proliferation, cell apoptosis inhibition, cell cycle acceleration, angiogenesis, and tumor metastasis (Jin et al., 2014). The PI3K/AKT/mTOR signaling pathway is mainly composed of PI3K, AKT and mTOR, which are frequently abnormal in OCCC, and any abnormality of the three can cause functional abnormalities of the PI3K/AKT/mTOR pathway (Caumanns et al., 2019). The *PIK3CA* gene (encoding the PI3K catalytic domain subunit) is most commonly altered in this pathway and is mutated in 30%–40% of OCCCs), whereas *PTEN* (antagonistic to PI3K) is not expressed in 40% of OCCCs (Hashiguchi et al., 2006; Kuo et al., 2009; Caumanns et al., 2018b). Other changes include deleterious mutations in *PIK3R1* (the regulatory subunit of PI3K) and amplifications and/or mutations of the PI3K downstream signaling molecules AKT1, AKT2, and AKT3 (Friedlander et al., 2016; Itamochi et al., 2017; Murakami et al., 2017).

PI3K is a group of membrane-associated lipid kinases composed of three subunits: the p85 regulatory subunit, p55 regulatory subunit and p110 catalytic subunit (Donahue et al., 2012). PI3K is mutated or overexpressed in a variety of cancers, including ovarian, breast, prostate, gastric, colorectal, glioblastoma, endometrial and brain cancers (Elmenier et al., 2019; Kato et al., 2019). Mutations in the *PIK3CA* gene, which encodes the P110 α protein in PI3K, lead to overactivation of PI3K kinase activity. *PIK3CA* gene mutations were found in 30%–40% of OCCC patients, and *PIK3CA* gene mutation was also found in endometriosis lesions of these patients, indicating that the PI3K-mediated pathway played an important role in the tumorigenesis of OCCC and that *PIK3CA* gene mutation is one of the early events in endometriosis-related OCCC (Kuo et al., 2009; Yamamoto et al., 2011; Murakami et al., 2021). Studies have shown that *PIK3CA* mutation is often seen in early OCCC, and patients harboring activated *PIK3CA* have longer OS and better prognosis than those without *PIK3CA* activation, possibly due to more indolent biological properties (Abe et al., 2013; Zannoni et al., 2014). Furthermore, it was reported that coexistent *ARID1A-PIK3CA* mutations could promote tumorigenesis of OCCC through protumorigenic inflammatory cytokine signaling (Chandler et al., 2015). In addition, the regulatory genes *PIK3R1* and *PTEN* were also inactivated in 40% of patients with *PIK3CA* activation. *PIK3R1* encodes the p85 regulatory subunit in PI3K, and its mutation can lead to downregulation, which occurs in 7%–8% of OCCCs. *PTEN* is a coregulatory factor in PI3K activity, and its deletion coordinates with *PIK3CA* gene mutation in tumorigenesis. *PTEN* deletion has been found in OCCC, ovarian endometrioid carcinoma and endometriosis (Sato et al., 2000; Kinross et al., 2012).

AKT, also known as protein kinase B (PKB), is a 60 kDa serine/threonine protein kinase and can be divided into three subtypes (AKT1, AKT2, AKT3 or PKBα, PKBβ, PKBγ) involved in various cellular activities, such as cell survival, proliferation, invasion, apoptosis and angiogenesis (Shariati and Meric-Bernstam, 2019). As a downstream protein of PI3K, AKT is a core protein kinase in the signaling pathway that can activate and regulate numerous downstream targets. AKT is activated in many precancerous lesions and cancers, and mutations and amplifications of AKT suggest poor prognosis (LoPiccolo et al., 2007). Increased copy numbers of *AKT2*, located at chr19q13.2, were reported in 24% of OCCCs (Oda et al., 2018). A study found *AKT2* amplification in OCCC with relatively shorter PFS by genomic analysis (Tan et al., 2011). Sasano et al. (2015) discovered that AKT was frequently activated in both early and late stages of OCCC, and the response to cyclophosphamide was more sensitive in cisplatin-resistant OCCCs whose AKT activation was also increased.

mTOR, a 289 kDa serine/threonine kinase, belongs to the PI3K-related protein kinase family and exists in the form of mTORC1 and mTORC2 complexes *in vivo*, which can regulate various basic cellular processes, including protein synthesis, cell growth, metabolism, senescence, regeneration and autophagy (Murugan, 2019). Numerous studies have found that mTOR is associated with a number of human diseases, such as cancers, neurodegenerative diseases and aging (Guo and Yu, 2019). Mabuchi et al. (2009) detected the expression of phosphorylated mTOR in 98 cases of primary ovarian cancer (52 cases of clear cell carcinoma and 46 cases of serous adenocarcinoma) by immunohistochemistry and showed that mTOR activation was higher in OCCC than serous ovarian cancer (86.6% vs. 50%) (Mabuchi et al., 2009). Another study also confirmed more activation of mTORC2 in OCCC than in serous ovarian cancer (Hisamatsu et al., 2013). In addition, studies have found that the level of phosphorylated mTOR in late OCCC was significantly higher than that in early OCCC (100% vs. 83.6%), indicating that the expression of phosphorylated mTOR in OCCC was significantly correlated with disease stage (Khemapech et al., 2012). However, the clinical trial of mTOR inhibitor temsirolimus has shown unsatisfactory effects in OCCC (Farley et al., 2016). Even so, another clinical trial of dual mTOR inhibitor TAK228 is still on-going (de la Rosa et al., 2022).

Activation of the PI3K/AKT/mTOR signaling pathway has become a research focus of OCCC and its chemotherapy resistance, and many signaling pathway-related inhibitors are in the stage of clinical trials. It is believed that with the deepening of studies on the regulatory mechanisms and effects of this pathway and the evolution of new inhibitors, targeted therapies through the PI3K/AKT/mTOR signaling pathway in OCCC will have far-reaching significance.

### 2.3 High-frequency amplification and overexpression of *MET*

**FIGURE 3:**
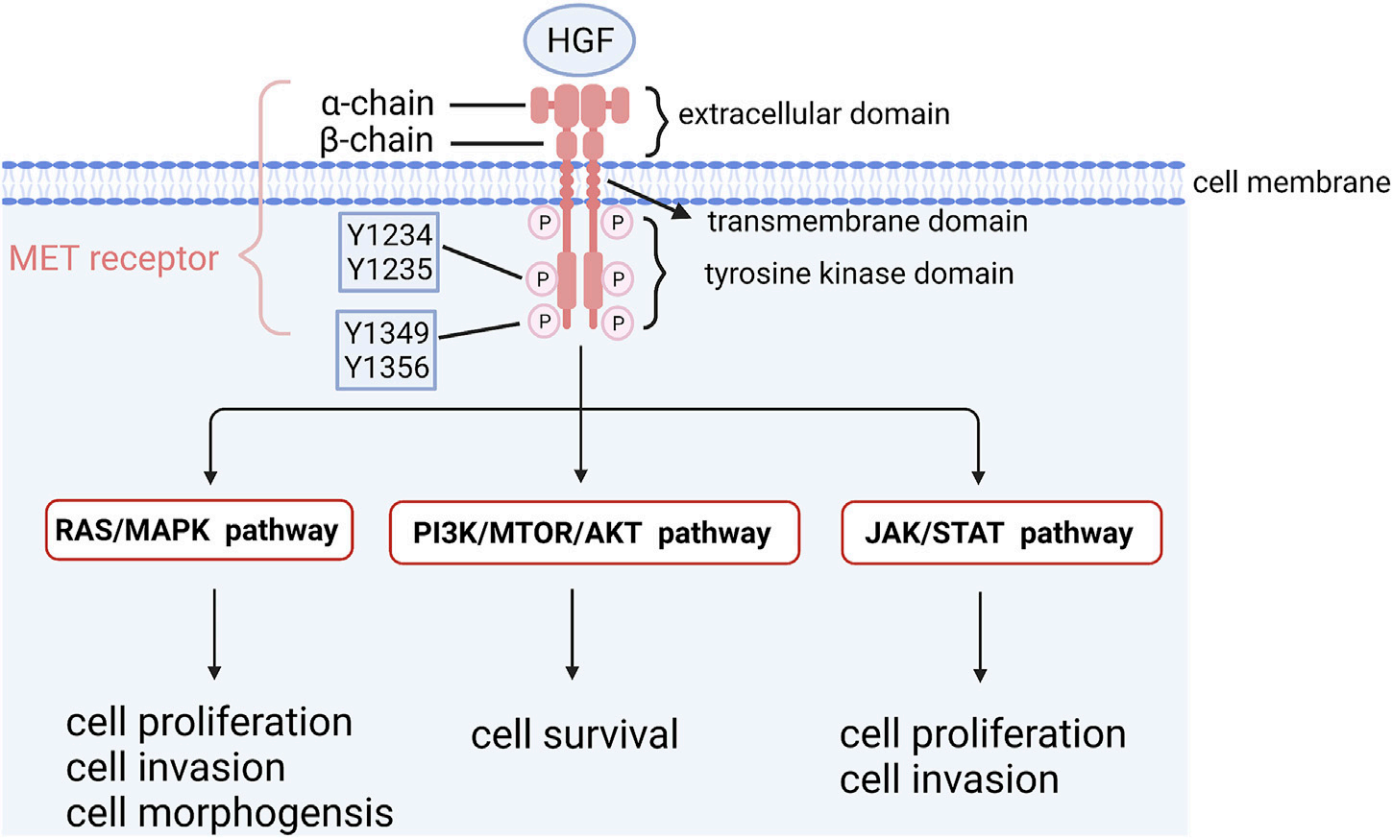
The structure of *MET* and its downstream signaling pathways. HGF binds to MET and regulates kinase activity by inducing homodimerization and phosphorylation of Y1234 and Y1235, two catalytic sites of tyrosine residues in MET receptor. Phosphorylated carboxyl terminal containing Y1349 and Y1356 serves as docking sites for intracellular protein kinases, transmits signals downstream and activates RAS-MAPK signaling pathway, PI3K/AKT/mTOR signaling pathway and other pathways to promote cell proliferation and survival.

Mesenchymal to epithelial transition factor (*MET*) is a proto-oncogene encoding MET kinase, which is a receptor of hepatocyte growth factor (HGF) on the cell surface (Cooper et al., 1984; Bottaro et al., 1991). MET kinase belongs to the receptor tyrosine kinase family and consists of three parts: extracellular parts (heterodimer binding α chain and *ß* chain by sulfide), transmembrane part (combination of the receptor signaling c-Cbl binding domain and structure domain near the membrane) and intracellular receptor tyrosine kinase domain (Eder et al., 2009). HGF binds to MET and regulates kinase activity by inducing homodimerization and phosphorylation of Y1234 and Y1235, two catalytic sites of tyrosine residues in the MET receptor. Phosphorylated carboxyl terminals containing Y1349 and Y1356 serve as docking sites for intracellular protein kinases, transmit signals downstream and activate the RAS-MAPK signaling pathway, PI3K/AKT/mTOR signaling pathway, JAK/STAT signaling pathway and other pathways to promote cell proliferation and survival (Longati et al., 1994; Furge et al., 2001; Hervieu and Kermorgant, 2018) (Figure 3). *MET* is expressed in epithelial cells of many organs during embryonic development and adulthood, including the liver, pancreas, prostate, kidney, muscle and bone marrow (Eder et al., 2009). However, *MET* is activated in tumor cells through gene amplification and overexpression, leading to a series of invasive growth processes in tumorigenesis, including cell proliferation, invasion and angiogenesis (Comoglio and Boccaccio, 2001; Boccaccio and Comoglio, 2006). Amplification of the *MET* gene has been found in various tumors, such as gastric, esophageal, lung and colorectal cancers (Michaels et al., 2018; Zhang et al., 2018; Seo et al., 2019; Gainor et al., 2020). The deletion of *MET* exon 14 skipping transcripts will lead to abnormal activation of downstream signaling pathways, which was not detected in OCCC yet. Compared with serous ovarian cancer and normal ovarian tissue, *MET* amplification and expression in OCCC were significantly increased (Kim et al., 2016). Yamashita et al. used real-time fluorescence quantitative PCR to analyze tumor tissues of 73 OCCC patients and found that 37.0% had *MET* gene amplification. The survival rate of stage I and II patients with *MET* gene amplification was significantly lower than that of patients without *MET* gene amplification. *MET* knockout with shRNA increased apoptosis and cell senescence, and the survival rate of OCCC cells with amplification of *MET* decreased (Yamashita et al., 2013). Wang et al. (2016) conducted immunohistochemistry (IHC) on the tissues of 86 OCCC patients and found that 94.2% of them had high *MET* expression, and the expression intensity of *MET* was closely related to chemotherapy resistance and poor prognosis. In addition, studies on endometriosis-related OCCC found that the incidence of *MET* overexpression increased with the tumorigenesis of OCCC: non-atypical precursor lesions (0%), atypical lesions (67%), relatively differentiated cancer components (92%), and poorly differentiated cancer components (100%), suggesting that accumulative *MET* gene copy number alterations causing *MET* overexpression are associated with higher tumor grade (Yamamoto et al., 2012b). These results demonstrated that *MET* overexpression was one of the early carcinogenesis events in *MET*-amplified OCCC and might promote the development of OCCC. Studies have found that dozens of MET inhibitors, such as INCB28060, PHA665752, HS10241, INC280, PF-2341066 and MK803, can inhibit ovarian cancer tumor growth and metastasis and reverse chemotherapy resistance (Zillhardt et al., 2010; Marchion et al., 2013; Moran-Jones et al., 2015; Li et al., 2016; Wang and Cheng, 2017; Han et al., 2019). The clinical trial NRG-GY001 demonstrated that the *MET* inhibitor cabozantinib had no clinical benefits for clear cell ovarian (Konstantinopoulos et al., 2018).

In summary, the high frequency amplification and overexpression of *MET* in tumors and its high specificity for OCCC suggest that *MET* is a good candidate gene for OCCC targeted therapy. The emergence of a large number of *MET*-related inhibitors in recent years provides the possibility of drug screening for OCCC targeted therapy.

### 2.4 Amplification of *HER2*

Human epidermal growth factor receptor 2 (*HER2*) is also one of the current research focuses. Amplification of *HER2* is closely related to chemotherapy resistance and poor prognosis of tumors. A study showed that *HER2* positivity by ISH (*in situ* hybridization) was 16.7% in OCCC (Koopman et al., 2018). Tan et al. (2011) found that *HER2* gene amplification and protein overexpression were observed in 14% of OCCCs, suggesting that *HER2* may be a potential therapeutic target for OCCC. However, in another study, different levels of *HER2* protein expression were detected by western blot in 10 OCCC cell lines, while *HER2* protein expression was only detected in 5 cell lines by IHC, and *HER2* gene amplification was only observed in RMG-1-cell lines with fluorescence *in situ* hybridization (FISH), indicating that *HER2* targeted therapy might benefit only a few OCCC patients (Itamochi et al., 2007). Fujimura et al. (2002) discovered that *HER2* was overexpressed in 42.9% OCCC by IHC and that three OCCC cell lines, RMG-1, HAC-II and KK, also showed positive staining for *HER2*, with expression levels 7.2, 6.4 and 4.5 times higher than those in normal mammary glands, respectively. Trastuzumab, as a human monoclonal antibody against *HER2*, significantly inhibited the growth of OCCC cell lines *in vitro* and RMG-1-transplanted tumor growth *in vivo* in a dose-dependent manner. The overall survival of trastuzumab-treated mice was also longer than that of the control group. A clinical trial on trastuzumab treating *HER2*-overexpressing ovarian and primary peritoneal cancers observed an overall response rate of only 7%. This might be the reason that the selected 41 patients (7 of whom were OCCC) were only tested for *HER2* expression by IHC, and the gene copy number of *HER2* was not evaluated (Bookman et al., 2003). Therefore, future studies on targeting *HER2* for OCCC may need to evaluate both the amplification of the *HER2* gene and protein overexpression level, as well as develop *HER2* inhibitors with stronger targeting effects or conduct research on drug combination therapy.

### 2.5 Expression of HNF-1β

Hepatocyte nuclear factor-1β (*HNF-1β*) is a transcriptional activator that regulates gene promoters and enhancers and is expressed in the liver, digestive tract, pancreas and kidney. A study revealed that *HNF-1β* was the most abundantly upregulated transcription factor in OCCC, with an expression array study among OCCC and non-OCCC cell lines (Yamaguchi et al., 2010). Studies have shown that *HNF-1β* is an effective molecular marker for endometriosis and OCCC tumors and is overexpressed in 40% of endometriotic cysts without malignancy and in almost all cases of OCCC (Kato et al., 2006). Another study also showed that *HNF1-β* is overexpressed in OCCC and useful in cases of diagnostic uncertainty (82.5% sensitivity and 95.2% specificity for OCCC vs. HGSOC) (Kobel et al., 2009). However, the role of *HNF-1β* in OCCC and endometriosis remains uncertain. A recent study revealed the novel HNF-1β/GSK-3β/p-NF-κB pathway in OCCC, occurring in response to DNA damage (Kawahara et al., 2020). In addition, another study found that *HNF-1β* interacted with the NF-κB signaling pathway, increasing cell survival by altering apoptotic events, especially by upregulating *Bcl-2* expression in OCCC *via* a mitochondria-mediated pathway (Suzuki et al., 2015). Furthermore, it was found that RNA interference could be used to reduce *HNF-1β* expression, which may lead to OCCC cell apoptosis (Kobayashi et al., 2009). The results suggest that HNF-1β overexpression is involved in inhibiting apoptosis in OCCC cells. However, no drugs targeting *HNF-1β* have been developed, and further studies on the regulatory mechanism of *HNF-1β* in OCCC are needed.

### 2.6 Overexpression of *AnxA4*

Annexin A4 (*AnxA4*), an important member of the annexin superfamily, binds to calcium ions and phospholipids simultaneously to regulate cell membrane permeability and cell membrane transport, mediating cell growth and apoptosis, and participating in tumor proliferation, invasion and metastasis, angiogenesis and chemotherapy resistance (Yao et al., 2016). Wang et al. (2017) found that *AnxA4* contained a Lewis Y structure, which could modify and enhance its interaction with NF-κB p50 and promote the malignant progression of OCCC. Studies have shown that *AnxA4* is overexpressed in OCCC and induces chemotherapy resistance by enhancing drug efflux (Kim et al., 2009; Kim et al., 2010). Another study confirmed that *AnxA4* promoted platinum-drug resistance through *ATP7A*, a copper and platinum transporter (Matsuzaki et al., 2014). Furthermore, it was reported that the repeats of the calcium binding site in the *AnxA4* protein could induce resistance to platinum drugs by increasing the intracellular chloride concentration (Morimoto et al., 2014). In conclusion, *AnxA4* is a potential therapeutic target for OCCC. However, no drug has been proven to effectively inhibit *AnxA4* expression. Therefore, the development of an effective *AnxA4* inhibitor in combination with platinum therapy may be a new treatment for OCCC.

### 2.7 Amplification of *ZNF217*

The zinc finger protein 217 (*ZNF217*) gene, in the amplicon on chromosome 20q13.2, encodes a Krupple-like transcription factor and is a candidate oncogene expressed in 20%–30% of primary human breast cancers, with overexpression correlating with poor prognosis (Vendrell et al., 2012). *ZNF217* overexpression can inhibit signaling events and promote tumor metastasis and chemotherapy resistance (Littlepage et al., 2012). *ZNF217* gene amplification was reported in 31% of OCCC cases, and protein overexpression was reported in 40% of OCCC cases (Huang et al., 2014). Rahman et al. demonstrated that *ZNF217* gene amplification was significantly associated with lymph node metastasis of OCCC. Compared with non-*ZNF217*-amplified cells, *ZNF217*-amplified cells showed stronger inhibition of cell migration and invasion after treatment with siRNA, suggesting that *ZNF217* might be a new therapeutic target for OCCC (Rahman et al., 2012).

### 2.8 Other genomic alterations in ovarian clear cell carcinoma

In addition to the mutated genes listed above, some other frequently mutated genes in OCCC were also identified by WES or targeted multiple gene panel testing, including *ARID1B* (AT-rich interactive domain 1B), *KRAS* (V-Ki-ras2 Kirsten ratsarcoma viral oncogene homolog), *PPP2R1A* (protein phosphatase 2 regulatory subunit A, alpha, encoding serine/threonine protein phosphatase 2 scaffold subunit alpha), *SMARCA4* (SWI/SNF-related, matrix-associated, actin-dependent regulator of chromatin, subfamily A, member 4, encoding ATP-dependent chromatic modeller BRG1), *MLL3* (mixed lineage leukemia 3), *ERBB2* (V-Erb-B2 avian erythroblastic leukemia viral oncogene homolog 2, also known as *HER2*), *TFAP2A* (transcription factor AP-2 alpha), *TP53* and *CTNNB1* (catenin beta 1, encoding betacatenin) (Tan et al., 2011; Arildsen et al., 2017; Itamochi et al., 2017; Maru et al., 2017; Murakami et al., 2017; Kim et al., 2018; Shibuya et al., 2018). Regarding the profiles of chromosomal CNVs in OCCC, although focal CNVs at specific gene loci were less frequent in OCCC than in HGSOC, recurrent CNVs were identified at various loci (Sung et al., 2013; Okamoto et al., 2015; Uehara et al., 2015; Mabuchi et al., 2016). In addition to *MET*, *AKT2*, and *ZNF217*, which are listed above, it was reported that amplification of chromosome 8 (8p11.21-q11.23 and 8q22.1-q24.13) was detected in 52% of OCCCs (Uehara et al., 2015), as well as copy number loss (loss of heterozygosity or homozygous deletion) at the loci of *CDKN2A/2B* (Cyclin-Dependent Kinase Inhibitor 2A/2B) (9p21.3) in 17% of OCCCs (Kuo et al., 2010). Other CNVs identified include amplification of chromosome 8q (64%), 17q (46%) and 20q (54%), and deletion of 9q (21%), 13q (28%), 18q (21%), and 19p (41%) by WES (Murakami et al., 2017). Furthermore, in addition to *HNF-1β*, multiple microarray datasets revealed additional significantly upregulated genes in OCCC, including *VCAN, HIF-1α, IL-6, p21, STAT3* and other genes related to oxidative stress and coagulation (Yamaguchi et al., 2010; Diaz et al., 2013; Matsuo et al., 2015).

## 3 Epigenomics of ovarian clear cell carcinoma

Epigenomics is the study of epigenetic modifications at the genome level. Epigenetic modifications act on DNA and histones in cells, including DNA methylation, DNA hydroxymethylation, posttranslational modifications of histones (acetylation, methylation, etc.) and chromatin remodelling. Epigenetic alterations in the genome have important effects on physiological processes and tumorigenesis.

### 3.1 Abnormal DNA methylation

DNA methylation is an epigenetic mechanism of gene regulation and plays an important role in various biological processes, such as X-chromosome inactivation, genomic imprinting, aging, and cancer (Houshdaran et al., 2010). Abnormal DNA methylation, either global DNA hypomethylation or aberrant hypermethylation of tumor suppressor genes, contributes to tumorigenesis (Gargiulo and Minucci, 2009). A variety of studies have identified that the OCCC methylation profile is distinct from the other histologic subtypes of ovarian cancer (Yamaguchi et al., 2014a; Yamaguchi et al., 2014b; Engqvist et al., 2020). Yamaguchi et al. collected genome-wide methylation and gene expression data for 14 OCCC, 32 non-OCCC and 4 non-cancerous cell lines and found inverse relationships between gene expression and methylation, including 22 hypomethylated genes and 276 hypermethylated genes, suggesting functional regulation by methylation. Furthermore, categorical and pathway analyses indicated that the hypomethylated genes were involved in the response to stress and the HNF-1β pathway and had increased expression, such as *HNF-1α, HNF-1β, PAX8* (paired box gene 8) and *SGK2* (serum and glucocorticoid kinase 2), while the hypermethylated genes included members of the estrogen receptor α (ERα) network genes involved in tumor development and had decreased expression (Yamaguchi et al., 2014b). Another study discovered that the low expression levels of *SLIT2* in OCCC were the result of promoter hypermethylation and were associated with tumor migration (Lin et al., 2021). It was reported that *MLH1* promoter hypermethylation could cause mismatch repair deficiency (MMRD) and MSI, leading to tumorigenesis in endometriosis-associated OCCC (Leskela et al., 2020). Additionally, methylation levels at the RASSF2A promoter were found to be obviously higher in endometriosis-associated OCCC than in ovarian endometriosis cyst and normal endometrial tissues (Xia et al., 2018). Senthong et al. (2014) indicated that hypomethylation of long interspersed element-1 (*LINE-1*) was an early molecular event participating in OCCC malignant transformation. Methylation of *HIN-1* is also associated with chemotherapy resistance and poor outcomes in OCCC (Ho et al., 2015). Several other genes have also been reported to be aberrantly methylated in OCCC, including 14-3-3σ, *TMS1/ASC* (TMS1 (Target of Methylation induced Silencing), also known as *ASC* (Apoptosis Speck like protein containing a CARD)), *WT1* (Wilms’ tumor 1), *RASSF1A* (ras association domain family 1A gene), *CDH13* (cadherin 13), *CACNA1A* (calcium voltage-gated channel subunit alpha1 A), *KCNH2* (the K (+) voltage-gated channel subfamily H member 2, also known as the human ether-a-go-go-related gene or hERG), *THBS2* (thrombospondin 2), *DLEC1* (deleted in lung and esophageal cancer 1), and *SFRP5* (secreted frizzled-related protein 5) (Terasawa et al., 2004; Asadollahi et al., 2010; Ho et al., 2012; Cicek et al., 2013; Kelemen et al., 2013; Chen et al., 2015). In short, abnormal DNA methylation regulates specific pathways and biological functions in OCCC, influences the characteristic biology and contributes to tumorigenesis.

### 3.2 Modification of histones

Histones bind to DNA to form chromatin in eukaryotes, and modification of histones, including methylation, acetylation, small ubiquitin-like modifications, phosphorylation, ubiquitination and so on, can regulate gene expression by histone modification enzymes, which play an important role in tumorigenesis (Esteller, 2007; Audia and Campbell, 2016). It was reported that *ARID1A* suppressed histone deacetylase 6 (*HDAC6*) in OCCC directly, and *HDAC6* nuclear expression was associated with immuno- and hypoxia tolerance and cancer stem cell phenotype in OCCC and upregulated following chemotherapy, leading to a poor prognosis (Yano et al., 2018; Yano et al., 2019). Additionally, *HDAC6* inhibition selectively was found to promote apoptosis of *ARID1A*-inactivated cells, suggesting the use of HDAC6 inhibition in the treatment of OCCC (Altucci, 2017). Expression of *HDAC 1* and *7* was also identified with a poor prognosis in OCCC (Yano et al., 2018). Approximately half of OCCC had mutations in the SWI/SNF chromatin remodelling complex subunit *ARID1A*, and cross-cancer analysis of the TCGA database showed that lysine-specific histone demethylase 1 (*LSD1*), which regulated the chromatin landscape and gene expression by demethylating proteins such as histone H3, was highly expressed in SWI/SNF-mutated tumors. Furthermore, the reversible *LSD1* inhibitor SP-2577 has the ability to promote antitumor immunity and T cell infiltration in OCCC cell lines (Soldi et al., 2020). Several studies have demonstrated synthetic lethality by targeting *EZH2* (enhancer of Zeste homolog 2) histone methyltransferase activity in *ARID1A*-mutated OCCC (Bitler et al., 2015). The results of another study suggested that the histone methyltransferase *SET* and *MYND* domain containing 2 (*SMYD2*) could promote OCCC viability and inhibit *SMYD2*-induced apoptosis in OCCC cells (Kojima et al., 2020). Wolf-Hirschhorn syndrome candidate gene-1 (*WHSC1*), a histone methyltransferase, has also been found to be upregulated in OCCC, leading to cell growth induction (Kojima et al., 2019). At present, there are relatively few studies on histone modification in OCCC. As the research goes further, novel molecular targeted approaches for OCCC treatment may be represented.

### 3.3 Chromatin remodelling complex

Chromatin remodelling complexes use the energy of ATP to move nucleosomes in the genome and play a crucial role in controlling chromatin structure and regulating gene transcription. According to their structure and function, chromatin remodelling complexes can be divided into four major groups: SWI/SNF, CHD (Chromodomain-Helicase DNA binding protein family), ISWI (Imitation SWItch family), and INO80 (INOsitol-requiring 80 family) (Clapier and Cairns, 2009). Except for *ARID1A* mutation leading to the inactivation of SWI/SNF, which we discussed above, it was reported that BRG1 phosphorylation inhibited the functions of the SWI/SNF complex in chromatin activation and thus derived tumor malignancy, thereby promoting the expression of various cancer-related proteins (Kimura et al., 2021). Furthermore, two other genes involved in chromatin remodelling, *KMT2D* (the lysine (K)-specific methyltransferase 2D gene) and *SPOP* (Speckle-type POZ protein), may also contribute to the tumorigenesis of OCCC (Arildsen et al., 2017). *SPOP* was reported to indirectly affect heterochromatin maintenance through ubiquitination of the *DAXX* protein in kidney cancer (Li et al., 2014). Meanwhile, the protein encoded by *KMT2D* is a histone methyltransferase that methylates the Lys-4 position of histone H3 and is part of a large protein complex called ASCOM (activating signal cointegrator-2 complex). ASCOM is recognized as a physiologically relevant coactivator for *p53* and is involved in the *p53* tumor suppression pathway, which may be significant for OCCC tumorigenesis (Lee et al., 2009). Importantly, mutations of *ARID1A*, *SPOP* and *KMT2D* were mutually exclusive (Arildsen et al., 2017).

## 4 Conclusion

As mentioned above, we summarized the gene alterations and associated clinical trials in ovarian clear cell carcinoma [Table 1, (Bookman et al., 2003; Farley et al., 2016; Miller et al., 2016; Alldredge and Eskander, 2017; Konstantinopoulos et al., 2018; de la Rosa et al., 2022)]. In summary, OCCC has distinct genomic and epigenomic profiles compared to other histologic types of EOC. With the help of clinical sequencing in OCCC, numerous types of genomic and epigenomic alterations have been identified, which may shed light on novel therapeutic options in OCCC. Although OCCC is a rare tumor in gynecological cancers, the molecular targets are similar to those of tumors in other organs, especially renal clear cell carcinoma. Thus, we hope our review will contribute to the development of precise medicine against OCCC.

**TABLE 1:** Summary of the gene alterations and associated clinical trials in ovarian clear cell carcinoma.

Genes	Type of alterations	Pathways	Drugs	Trials	References
*ARID1A, ARID1B*	Mutation	ARID1A synthetic lethality	Dasatinib, EZH2 inhibitors (e.g., tazemetostat)	GOG 283	Miller et al. (2016), Alldredge and Eskander (2017)
*PIK3CA, PIK3R1, AKT, mTOR*	Amplification and mutation	PI3K/AKT/mTOR pathway	mTOR inhibitor (e.g., temsirolimus, TAK228)	GOG 268, NCT03648489	Farley et al. (2016), de la Rosa et al. (2022)
*HER2*	Amplification		Trastuzumab	Not mentioned	Bookman et al. (2003)
*MET*	Amplification and overexpression	HGF/MET pathway	Cabozantinib	NRG-GY001	Konstantinopoulos et al. (2018)
*HNF-1β*	Hypo-methylation and overexpression	HNF-1β/GSK-3β/p-NF-κB pathway	—	—	Suzuki et al. (2015)
*AnxA4*	Overexpression	NF-κB p50	—	—	Wang et al. (2017)
